# Differences in within‐plant oviposition preferences and immature survival between *Orius* predators and the importance of spatial availability of prey

**DOI:** 10.1111/1744-7917.13465

**Published:** 2024-10-31

**Authors:** Angelos Mouratidis, Christiaan Bootsma, Marcel Dicke, Gerben J. Messelink

**Affiliations:** ^1^ Business Unit Greenhouse Horticulture & Flower Bulbs Wageningen University & Research Bleiswijk the Netherlands; ^2^ Laboratory of Entomology Wageningen University & Research Wageningen the Netherlands

**Keywords:** biological control, omnivore, *Orius laevigatus*, *Orius majusculus*, oviposition, preference‐performance

## Abstract

Oviposition preferences of plant‐feeding predators remain a complex topic, as such omnivores choose oviposition sites by assessing both plant characteristics and the quality and quantity of nearby animal food sources. *Orius* predators are omnivores that oviposit endophytically, thus plant characteristics play an important role in their oviposition choices. In this study, we assessed the oviposition and foraging preferences of *O. laevigatus* and *O. majusculus* on vegetative and flowering chrysanthemum plants, and assessed the survival of their offspring on differently aged tissues. Our results show a preference of *O. laevigatus* for young and tender chrysanthemum tissues, where the survival of the nymphs was longer on a plant diet. In contrast, *O. majusculus* selected older plant parts when laying its eggs, and nymphs did not survive long on any of the plant tissues offered. The foraging activity of *Orius* females for animal prey (*Ephestia kuehniella* eggs) did not reveal any specific pattern for either of the two predators. Furthermore, we tested the plasticity of the within‐plant oviposition preferences of *O. laevigatus*, by offering sentinel prey (*E. kuehniella* eggs) on distinct plant parts. We found that more eggs were laid in older plant tissue when animal prey was offered lower on the plant. Overall, our findings show that oviposition choices of *Orius* predators are based on a dynamic interplay between plant characteristics, presence of animal and/or floral food sources among other factors, and that differences may well occur between closely related species based on the importance of plant resources in their diet.

## Graphical abstract

*Orius* predators oviposit endophytically, and plant characteristics play an important role in their oviposition choices. In this study, we show that oviposition preferences differ between species, and that these differences may correlate with juvenile performance. Furthermore, we show that the within‐plant oviposition preference of *Orius* can be influenced by the spatial availability of food sources.

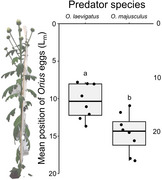

## Introduction

Insects typically forage in the habitat where their preferred food source can be found (Pyke *et al.*, 1977). When both adults and immature stages feed on the same plant or animal food sources, optimal foraging may overlap with the preference‐performance hypothesis, whereby females lay their eggs in proximity to the preferred food source (Jaenike, 1978; Scheirs & De Bruyn, 2002; Seagraves, 2009; Gripenberg *et al.*, 2010; Lundgren, 2011; Martínez *et al.*, 2013). These oviposition preferences are further typically thought to be weighed against the vulnerability of offspring to predation or parasitism, among other biotic and abiotic factors (Schellhorn & Andow, 1999; Seagraves, 2009; Gripenberg *et al.*, 2010). Many insects however consume both plant and animal food sources, thus these omnivores need to balance their oviposition decisions based on the spatial and temporal availability of both these resources (Coll & Guershon, 2002; Lundgren, 2009). Furthermore, several omnivorous predators exhibit endophytic oviposition, thus they need to locate suitable plant structures or sites that allow for the successful insertion and maturation of their eggs (Constant *et al.*, 1996; Lundgren *et al.*, 2008; Lundgren, 2011). Mirids, nabids, and anthocorids are omnivorous predators that insert their eggs in plant tissue, and their oviposition behavior and preferences have largely been studied due to their importance as biological control agents (Isenhour & Yeargan, 1982; Constant *et al.*, 1996; Sánchez *et al.*, 2004; Lundgren & Fergen, 2006; Lundgren *et al.*, 2008).

Minute pirate bug species of the genus *Orius* Wolff (Hemiptera: Anthocoridae) occur around the world (Lattin, 1999; Schuldiner‐Harpaz & Coll, 2022), and are important biological control agents of thrips (van den Meiracker & Ramakers, 1991; Funderburk *et al.*, 2000), aphids (Rutledge & O'Neil, 2005), but also feed on other small arthropods (Montserrat *et al.*, 2000; Venzon *et al.*, 2002). As omnivorous predators, they also consume plant resources, such as plant vascular sap (Armer *et al.*, 1998; Zeng & Cohen, 2000), (extra)floral nectar (Yokoyama, 1978; Pumariño *et al.*, 2012), and pollen (Vacante *et al.*, 1997). In fact, in many cases their reproduction increases when feeding on a mixed plant and animal diet (Cocuzza *et al.*, 1997; Pumariño & Alomar, 2012). *Orius* insert their ∼5.5 mm long oval shaped eggs inside plants, and are known to prefer plants and plant tissues with a thin epidermal layer and low trichome density (Armer *et al.*, 1999; Lundgren & Fergen, 2006; Lundgren *et al.*, 2008; Waite *et al.*, 2014; Zhang *et al.*, 2021). Furthermore, oviposition choices of *Orius* predators rely on proximal cues rather than infochemicals (Lundgren *et al.*, 2009). They tend to avoid laying their eggs in too thin tissue such as the leaf lamina, where the egg is not fully enclosed (Lundgren & Fergen, 2006; Pascua *et al.*, 2019; Zhang *et al.*, 2021), which may lead to low hatch rates (Groenteman *et al.*, 2006). Instead, anthocorids prefer to lay their eggs in succulent and turgid plant parts (Evans, 1976; Groenteman *et al.*, 2006), thus reducing the risk of egg mortality due to desiccation (Richards & Schmidt, 1996). Efforts to create artificial oviposition substrates, also found the thickness of paraffin‐coated gelatine structures to influence the acceptability for oviposition (Castañe & Zalom, 1994). Within‐plant site selection for oviposition by *Orius* predators thus depends on the site's characteristics and correlates with successful egg hatching (Lundgren *et al.*, 2008), yet it does not seem to be directly affected by the proximity of food sources (Coll *et al.*, 1997), similar to other insects of which the juveniles are highly mobile and can migrate to sites where food is more accessible after hatching (Martínez *et al.*, 2013). Nevertheless, previous research showed that for many *Orius* species oviposition in the proximity of flowers is preferred (Tawfik & Ata, 1973; van den Meiracker & Sabelis, 1993; Mouratidis *et al.*, 2023a), yet whether this preference is due to the suitability of the plant tissues themselves or due to their proximity to the floral resources is unclear.

Most previous research on within‐plant oviposition preferences and behavior of *Orius* predators has been conducted as single‐species studies (Isenhour & Yeargan, 1982; van den Meiracker & Sabelis, 1993; Lundgren *et al.*, 2008; Pascua *et al.*, 2019), thus not providing a broader fundamental understanding of this behavior and its variation among different species. In this study, we first evaluated the within‐plant oviposition and foraging preferences of *Orius laevigatus* (Fieber) and *Orius majusculus* Reuter, to determine whether differences between closely related predators of this genus exist. Experiments were conducted on potted chrysanthemum, as this vertically growing plant allows for detailed differentiation among plant strata with different anatomical characteristics. Both vegetative and flowering plants were used in our experiments, as we hypothesized that the presence of floral resources may affect the behavior of these omnivorous predators. Additionally, we evaluated the plasticity of the oviposition behavior in *O. laevigatus*, by providing an animal food source on distinct spatial strata. Finally, we assessed the survival of immature predator stages (eggs and nymphs) on the distinct parts chosen as oviposition sites by the females, aiming to correlate female oviposition preference to offspring performance.

## Materials and methods

### Plants and insects

Chrysanthemum plants (*Dendranthema grandiflora* Tzvelev, cv. Baltica) were used for all experiments. Plants were received from a commercial breeder (Deliflor, Maasdijk, the Netherlands) as cuttings rooted in soil cubes with three fully expanded leaves. Then, they were transplanted into plastic pots (10 cm × 10 cm) in potting soil and irrigated with a standard nutrient solution for young plants until use in experiments. In all plant propagation and experimental greenhouse compartments, temperature and relative humidity were registered every 5 min with a climate recorder (Hoogendoorn Growth Management). Plants were grown free of pesticides in a clean greenhouse compartment at 20.2 °C (range 17.6–22.9 °C) and average RH 68% (range 64%–79%). The light regime in the first 2 weeks of growth was 16 L : 8 D, and in the third week after transplanting it was altered to 11.5 L : 12.5 D in order to induce chrysanthemum flowering, according to agronomical practices. Plants were sprayed three times with the plant growth inhibitor daminozide (Dazide Enhance^®^, Royal Brinkman’s‐Gravenzande, the Netherlands). Daminozide inhibits the production of natural growth hormones and leads to increased plant vigor, darker leaf color and short and sturdy flower stems, limiting vertical growth (Rademacher, 2000). The product was applied in the third, fourth and fifth week after transplanting, with concentrations of 0.15, 0.2, and 0.25 g/L, respectively, according to standard practice and the manufacturer's recommendations. In all experiments, vegetative (6‐week‐old) or flowering (8‐week‐old) plants were used.

*Orius* predators used in all experiments originated from synchronized cultures (Mouratidis *et al.*, 2023b). Female adults of *O. laevigatus* and *O. majusculus* were obtained from Koppert Biological Systems B.V. (Berkel en Rodenrijs, the Netherlands) and EWH BioProduction (Tappernøje, Denmark), respectively. All predators were reared on flat green bean pods (*Phaseolus vulgaris* L.) and fed *ad libitum* with frozen UV‐irradiated *Ephestia kuehniella* Zeller (Lepidoptera: Pyralidae) eggs (Koppert B.V.) and *Artemia franciscana* Kellogg (Anostraca: Artemiidae) decapsulated cysts (BioBee Biological Systems, Sde Eliyahu, Israel). Cultures were kept in climate chambers (Economic Lux ECL02, Snijders Labs, Tilburg, the Netherlands) at 25 ± 1 °C, 70% ± 10% RH, and 16 L : 8 D.

### Within‐plant oviposition preference

Vegetative (6‐week‐old; ∼55 cm tall; 23 ± 2 fully expanded leaves) or flowering (8‐week‐old; ∼65 cm tall; 29 ± 2 fully expanded leaves; 7 ± 1 flowering side shoots counted from the plant's apex) chrysanthemum plants were used to assess the within‐plant oviposition preference of *Orius* predators. Plants were placed individually in insect‐proof cages (70 cm × 70 cm × 115 cm, BugDorm‐2400F, MegaView Science Co. Ltd.; Taichung, Taiwan, China) and provided with standard nutrient solution via a dripping irrigation system. The plant stem was supported by a wooden stick (60 cm high). A strip of *Artemia* cysts (4 cm wide, of which 0.5 cm was covered with cysts, BioArtFeed, BioBee Ltd.) was attached vertically across the plant stem and wooden support using hairclips, assuring the presence of a surplus animal food source across the plant canopy (Fig. S1). Then, 8 mated 1‐week‐old female predators were released in the cages at the base of the plant. Three days later, all predators were removed from the plants and the stem was harvested and transported to the lab, where the number and position of eggs on each internode (stem or leaf) were counted under a stereo microscope. Internodes were numbered sequentially, with 1 being the youngest internode with a fully expanded leaf. Two separate experiments were conducted, one with vegetative plants and another with flowering plants. In both experiments, the oviposition preferences of the two predator species, *O. laevigatus* and *O. majusculus*, were evaluated. Each treatment included at least six replicates. The average temperature was 22.5 °C (range 20.3–24.9 °C) and average RH was 66% (range 58%–72%).

The weighted mean internode positions where *Orius* females oviposited were calculated for each plant using the formula proposed by Perring *et al.* (1987): $L_{mean}=\sum l_{i}m_{i}/\sum m_{i}$, where $\;L_{mean}$ is the mean internode position where *Orius* eggs were found, $l_{i}$ is the internode position on the plant (with *i* = 1, 2, 3, … *n*, with 1 being the youngest internode with a fully expanded leaf), and $m_{i}$ the number of eggs found on each internode.

Data were initially assessed visually on whether they met the normality and homoscedasticity assumptions, and then analyzed with an ANOVA, followed by Tukey's HSD *post hoc* pairwise comparison. Statistical analyses were performed using R 4.2.2 (R. Core Team, 2021).

### Within‐plant predation/foraging activity

To assess the within‐plant foraging and predation preference of *Orius* predators, an experiment where animal sentinel prey was placed in different spatial strata of chrysanthemum plants was conducted. Sentinel predation cards were prepared on green carton paper, and double‐sided adhesive tape was applied on a 10.6 mm × 10.6 mm area, allowing the attachment of ∼550 *E. kuehniella* eggs, according to the methodology described by Mouratidis *et al.* (2023b). Vegetative (6‐week‐old) or flowering (8‐week‐old) chrysanthemum plants were divided into three equal strata along their stem, namely the low, middle, and high stratum (of about ∼18 ± 2 cm for vegetative, and 22 ± 2 cm for flowering plants). On the median leaf of each defined stratum the predation cards were placed and fixed with a small plastic clothespin, making food sources at all three spatial levels simultaneously available. Plants were placed in insect‐proof cages in a greenhouse compartment. A group of eight 1‐week‐old *O. laevigatus* or *O. majusculus* females were then released on the base of the plants. Three days later, the predation cards were removed and the number of predated eggs was counted using a computer algorithm previously trained through deep‐learning to identify predated *E. kuehniella* eggs (Mouratidis *et al.*, 2023b). Control predation cards were also placed on caged plants where no predators were released, but as the natural collapse rate of moth eggs did not exceed 5% of the total number of eggs per card, these were not included in the analysis. The experiment was replicated eight times. During the experiments, the average temperature was 20.1 °C (range 17.9–26.1 °C) and average RH of 76% (range 57%–82%).

The proportion of predated eggs per stratum and cage was calculated by dividing the number of eggs predated on each stratum by the total number of predated eggs per cage. Proportions were then analyzed separately for each predator and plant stage with linear mixed effects models (LME) with strata (upper, middle or lower) as the fixed factor and cage (plant) as the random factor.

### Effect of spatial availability of animal food on within‐plant oviposition preference

In order to assess the importance of spatial availability of an animal food source on the within‐plant oviposition preference of an *Orius* predator, an additional experiment was conducted. Flowering chrysanthemum plants (8‐week‐old) were prepared and placed in cages following the methodology described in the previous section. However, instead of attaching the food source along the plant stem, predation cards holding ∼550 *E. kuehniella* eggs (prepared according to the methods described in Mouratidis *et al.*, 2023b) were placed on (i) the 5th highest fully expanded leaf, (ii) the 5th lowest expanded leaf, or (iii) off‐plant on the cage wall at a height of ∼30 cm. Only *O. laevigatus* was evaluated here, as this species preferred ovipositing on younger plant tissues in the previous experiment, thus allowing us to test whether oviposition may be shifted toward the lower plant stratum. Eight 1‐week‐old *O. laevigatus* females were released at the base of plants and allowed to oviposit for 3 d. Plants were then harvested, transferred to the lab, the number of eggs found on each internode was scored, and the weighted mean internode position for each plant was calculated, as described in the previous section. Eight replicates of each treatment were conducted. The average temperature during the experiment was 19.6 °C (range 17.9–25 °C) and average RH of 71% (range 55%–78%). The weighted mean internode position of *O. laevigatus* eggs was calculated as described above (Perring *et al.*, 1987), and data were analyzed with an ANOVA, followed by Tukey's HSD *post hoc* pairwise comparison.

### *Orius* fecundity and fertility on differently aged plant stems

To assess the possible effect of plant tissue on oviposition and egg hatch rate of *Orius* predators, a laboratory experiment was conducted. Plant stems from flowering chrysanthemum plants were collected, and a 7 cm section was made 5 cm from the apex of the plant (young stem) or 5 cm from its base (old stem). Leaves on stem sections were removed and abscission wounds were covered with Parafilm^®^ to prevent oviposition on the wounded tissues, and as previous results showed a high preference for oviposition on the stem itself rather than leaves. Stem sections were placed vertically in moist floral foam blocks (4 cm × 4 cm × 4 cm, Oasis^®^, Smither‐Oasis North America, USA). Stems were then placed in plastic jars (Ø 11 cm × 13 cm) with lids drilled and covered with a fine mesh gauze for ventilation. *O. laevigatus* and *O. majusculus* adults (< 24 h since the final molt) were collected from the culture, and a single couple (male and female) was released in each arena. If a male predator died, a new young male from the culture was introduced. When a female died or escaped before the end of the experiment (which occurred in fewer than 5% of the tested females), the replicate was removed from further analyses. Predators were provided with *E. kuehniella* eggs as an *ad libitum* food source at the beginning of the experiment and refreshed every 3 d. Stem sections were initially changed after 7 d, as the pre‐oviposition period of females and the egg stage of these predators lasts about 3.5 and 4.5 d at 25 °C, respectively (Tommasini *et al.*, 2004; Mouratidis *et al.*, 2022), thus no eggs would hatch in this period. Thereafter, stem sections were changed two additional times at 3‐d intervals, thus oviposition was scored for 14 d of the females’ lifetime. Stems were examined and scored for eggs under the stereomicroscope. To evaluate the egg hatch rate, stem sections collected during the second stem change were incubated under the same conditions, and the emerging nymphs were counted and removed daily for three consecutive days. At least 9 replicates of each treatment were carried out for each predator, and the experiment was conducted in a climate chamber at 25 ± 1 °C, 70% ± 10% RH, and 16 L : 8 D.

Data on fecundity were initially assessed visually through residual diagnostic plots for compliance with a normal distribution and homoscedasticity, and then analyzed with ANOVA. Hatch rate dates were analyzed with a GLM with binomial distribution and a logit link function, accounting for overdispersion by including a dispersion parameter equal to Pearson's *χ* ^2^‐based dispersion divided by the residual degrees of freedom (Hardin & Hilbe, 2018).

### Nymphal survival on different plant food sources

To test potential differences in fitness of immature predators from feeding on different plant resources, a nymphal survival experiment was conducted. Stems were collected from 8‐week‐old flowering chrysanthemum plants (stem length ∼65 cm), and the stem was divided into three equal parts, upper, middle, and lower. Then, a ∼5 cm cut from the middle of each section was made, containing 1 fully expanded leaf. These plant sections were placed in 1.5 mL Eppendorf^®^ tubes filled with water to prevent plant tissue desiccation during the experiment. Leakage was prevented by sealing the tubes with Parafilm®. First‐instar nymphs (< 24 h old) of *O. laevigatus* or *O. majusculus* were placed individually in plastic cups (Ø 8 cm × 6 cm) with the lid drilled and covered with fine mesh for ventilation. Developing nymphs had access to chrysanthemum stems and leaves from the upper, middle or lower plant sections, but not to animal prey. Nymphal survival was checked every day until adulthood or death of the individual. Plant sections were refreshed every other day. In total six treatments were realized, and each predator‐diet combination was replicated at least 10 times, and the experiment was incubated in climatic chambers (25 ± 1 °C, 70% ± 10% RH, and 16 L : 8 D). A Cox regression model was initially fitted, including predator (*O. laevigatus* and *O. majusculus*), stem (upper, middle and lower), and their interaction as covariates; however, the model assumption (tested through Schoenfeld residuals) was not met. Thus, Kaplan–Meier survivorship curves were fitted for each species separately, and survival time was compared among treatments with log‐rank tests.

## Results

### Oviposition preference in plants with food availability across all spatial levels

**Fig. 1:**
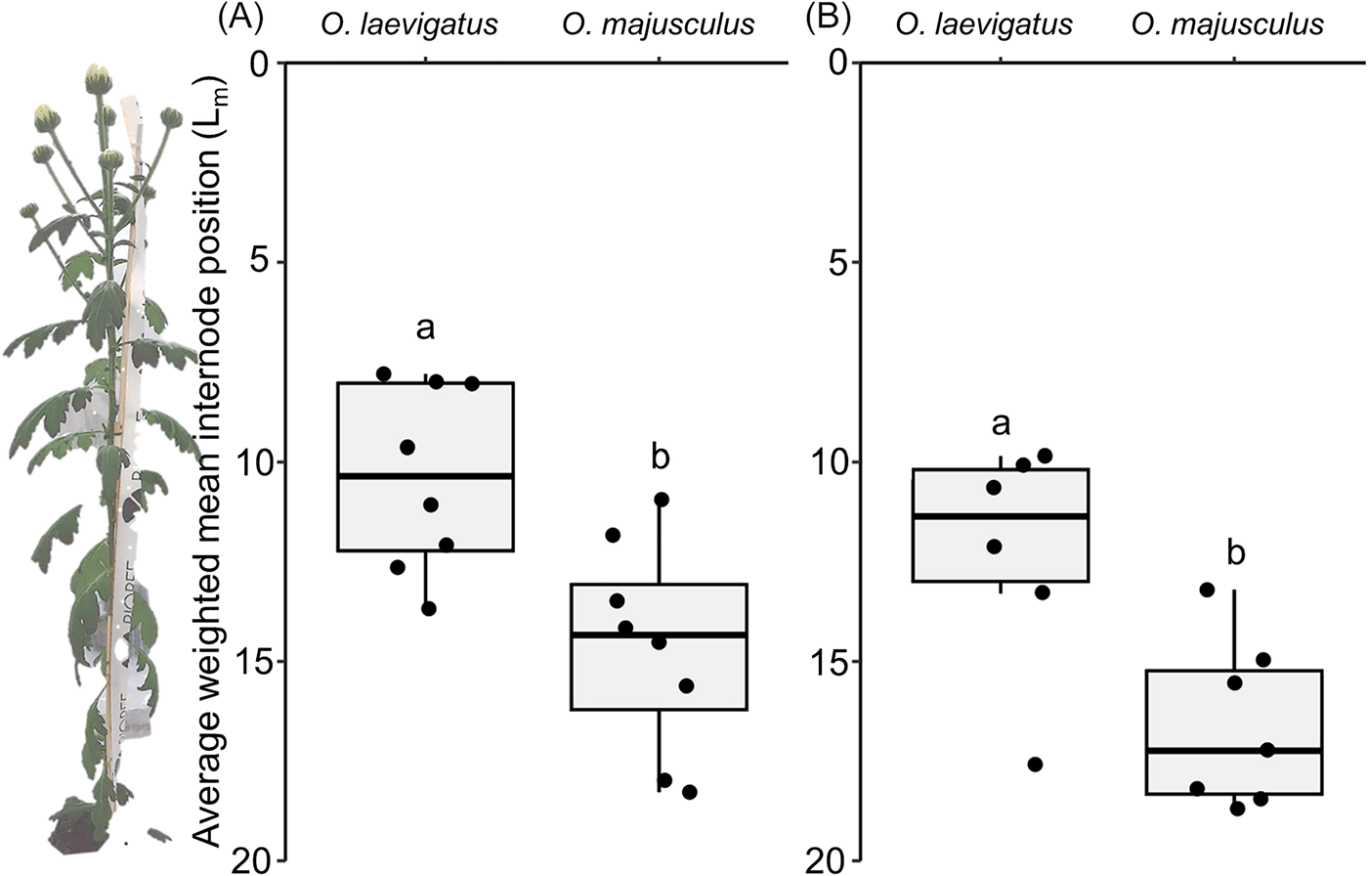
Average weighted mean leaf position (*L_m_*) of *Orius* eggs on (A) vegetative, and (B) flowering plants. Boxplots present mean quartiles and interquartile range, scatter depicts average weighted egg position within‐plant for each predator species. The numbering of leaves and internodes (*Y*‐axis) begins from the top of the plant with the youngest tissue. Bars marked with different lowercase letters within each panel are significantly different (ANOVA, Tukey's HSD, *P* < 0.05).

Differences in within‐plant oviposition between *Orius* predators were found on both vegetative (*F*_1, 14_ = 11.64, *P =* 0.004; Fig. 1A) and flowering plants (*F*_1, 11_ = 9.83, *P =* 0.009; Fig. 1B). Overall, *O. laevigatus* preferred to oviposit on younger tissue higher on the plant compared to *O. majusculus*.

### Within‐plant predation

**Fig. 2:**
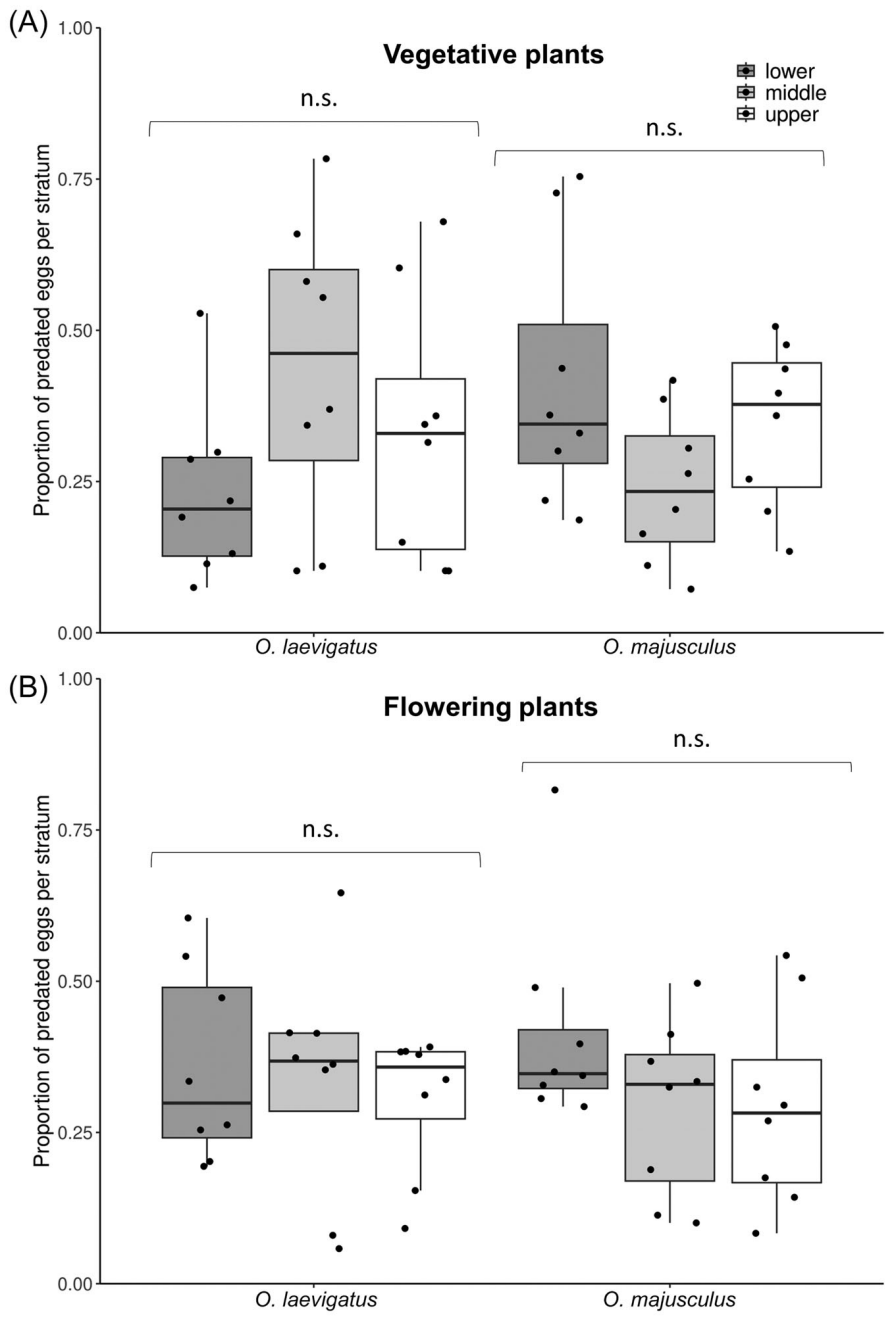
Proportion of *Ephestia kuehniella* eggs predated by *Orius laevigatus* and *O. majusculus* on (A) vegetative and (B) flowering chrysanthemum plants. Boxplots present mean quartiles and interquartile range, scatter depicts total proportion of eggs predated on each stratum by a group of predators (LME, n.s. means *P* > 0.05).

Both *O. laevigatus* and *O. majusculus* females consumed a similar proportion of eggs across the different plant strata in both vegetative (LME; *O. laevigatus*: *χ* ^2^ = 4.14, df = 1, *P* = 0.126; *O. majusculus*: *χ* ^2^ = 4.73, df = 1, *P* = 0.094; Fig. 2A) and flowering plants (LME; *O. laevigatus*: *χ* ^2^ = 0.54, df = 1, *P* = 0.764; *O. majusculus*: *χ* ^2^ = 3.33, df = 1, *P* = 0.189; Fig. 2B).

### Effect of spatial availability of prey on within‐plant oviposition preference

**Fig. 3:**
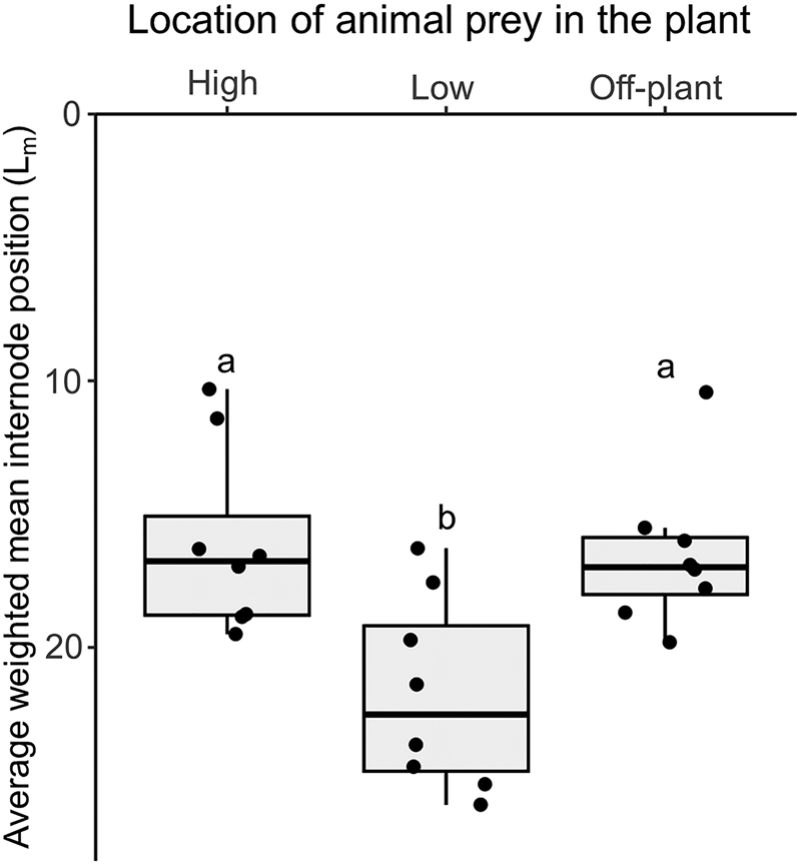
Average weighted mean leaf position (*L_m_*) of *Orius laevigatus* eggs on flowering chrysanthemum plants where animal prey (*E. kuehniella* eggs) was offered on the 5th youngest expanded leaf (high), on the 5th oldest expanded leaf (low), or off‐plant attached to the cage wall. Boxplots present mean quartiles and interquartile range; scatter depicts average weighted egg position within‐plant for each predator species. The numbering of leaves and internodes (*Y*‐axis) begins from the top of the plant with the youngest tissue. Bars marked with different lowercase letters are significantly different (ANOVA, Tukey's HSD, *P* < 0.05).

The spatial availability of sentinel animal prey had a significant effect on the within‐plant oviposition preference of *O. laevigatus* (*F*_2, 21_ = 7.3, *P =* 0.004). When sentinel prey was placed on the 5th oldest expanded leaf, significantly more eggs were found on lower and older plant structures, compared to the treatment where sentinel prey was offered on the 5th youngest leaf or off‐plant (Fig. 3).

### Fecundity and fertility on differently aged plant stems

**Fig. 4:**
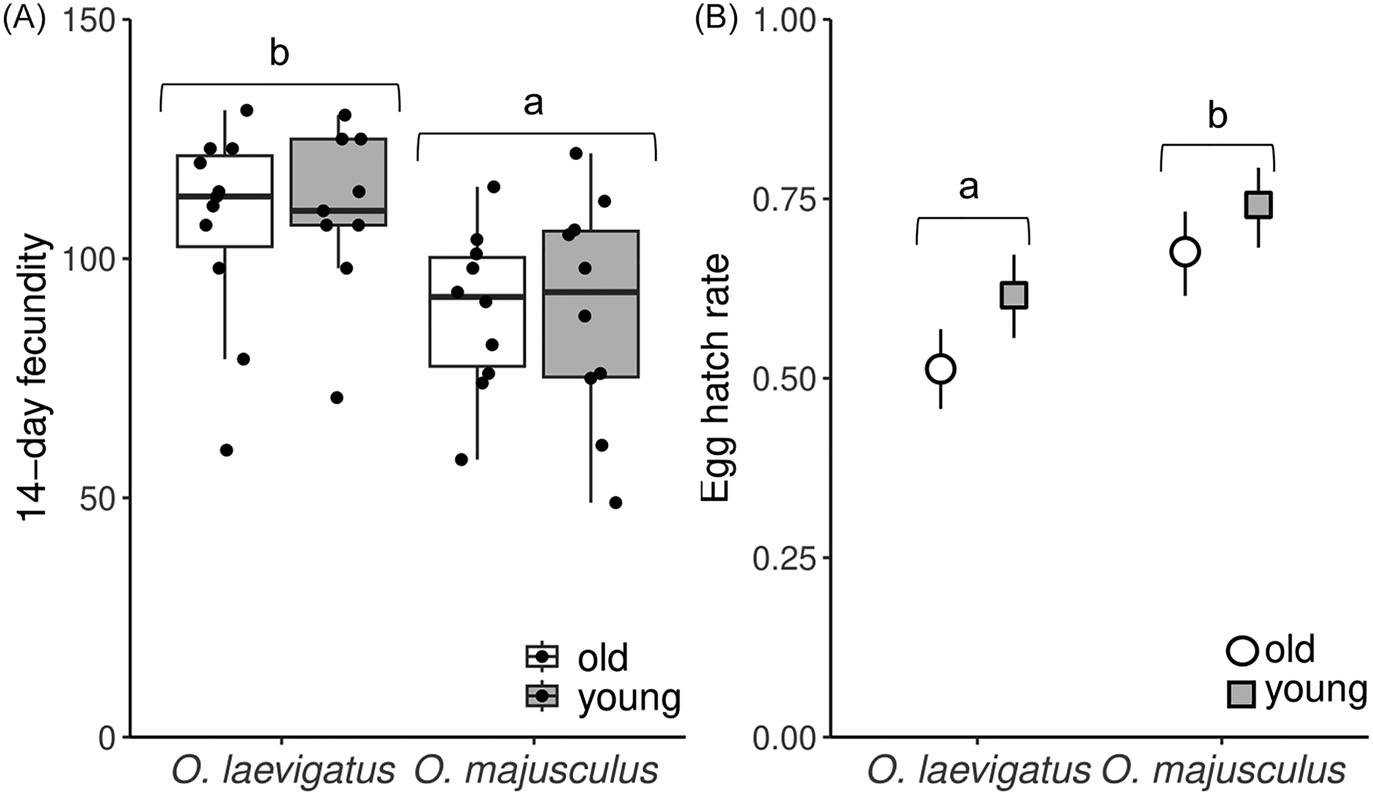
(A) Fecundity and (B) egg hatch rate (mean proportion ± 95% CI) of *Orius laevigatus* and *O. majusculus* on young and old chrysanthemum stem tissue. Boxplots present mean quartiles and interquartile range; scatter depicts total number of eggs laid by each experimental individual in respective treatments. Treatment groups marked with different lowercase letters are significantly different (Fecundity: ANOVA; Egg hatch rate: GLM, *P* < 0.05).

Oviposition of *O. laevigatus* and *O. majusculus* was assessed for 2 weeks on chrysanthemum stems of different ages. *Orius laevigatus* laid significantly more eggs than *O. majusculus* (*F*_1, 36_ = 9.04, *P* = 0.005), but stem age did not significantly affect fecundity (*F*_1, 36_ = 0.038, *P* = 0.847), nor for the interaction between stem age and *Orius* species (*F*_1, 36_ = 0.038, *P* = 0.846; Fig. 4A).

A greater percentage of *O. majusculus* eggs hatched across both tissues compared to *O. laevigatus* (*χ* ^2^ = 24.66, df = 1, *P* = 0.01). Neither stem age nor the interaction between stem age and *Orius* species influenced the egg hatch rate significantly (stem age: *χ* ^2^ = 8.46, df = 1, *P* = 0.132; interaction: *χ* ^2^ = 0.15, df = 1, *P* = 0.841; Fig. 4B).

### Nymphal survival on different plant food sources

**Fig. 5:**
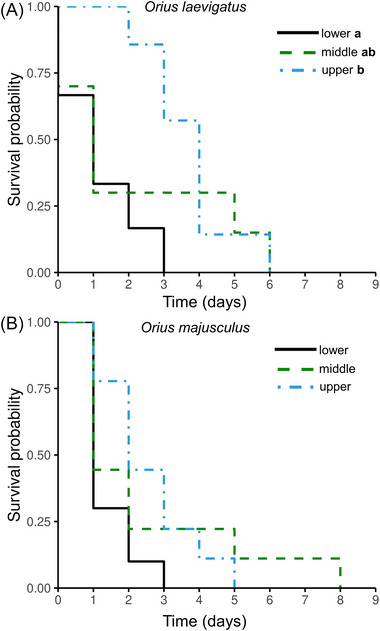
Cumulative proportion of surviving neonate nymphs for (A) *O. laevigatus* and (B) *O. majusculus*, on chrysanthemum stem sections with leaves from the lower, middle, or upper plant part. Different letters next to the legend indicate significant differences among treatments (Kaplan–Meier, *P <* 0.05).

Survival of *O. laevigatus* nymphs differed among the plant parts tested (*χ* ^2^ = 7.3, df = 2, *P* = 0.03), with individuals showing the highest survival on the youngest chrysanthemum stems (Fig. 5A). In contrast, the survival of *O. majusculus* nymphs was not influenced by the age of the chrysanthemum stems (*χ* ^2^ = 3.7, df = 2, *P* = 0.2; Fig. 5B).

## Discussion

Our study shows that within‐plant oviposition preferences can differ between closely related *Orius* predators. *Orius laevigatus* showed a preference for oviposition in younger plant tissue, in both vegetative and flowering chrysanthemum plants, compared to *O. majusculus*. Most of the eggs were laid in the plant's main stem, adjacent to the leaf petioles and axils. This site was probably chosen over the petioles or the leaf veins due to its lower density of trichomes (Lundgren *et al.*, 2008; Zhang *et al.*, 2021), while also providing cover for the ovipositing females, as *Orius* are known to preferentially reside in narrow sites with cover (Chambers *et al.*, 1993; van den Meiracker & Sabelis, 1993). Older stem tissue has a thicker epidermis, thus greater force would be needed to successfully insert eggs. The bigger size of *O. majusculus* compared to *O. laevigatus* (Péricart, 1972; Mouratidis *et al.*, 2022) may have allowed it to accept a wider range of plant epidermal thickness as acceptable oviposition substrate. Furthermore, the duration of oviposition may vary among plant substrates, and was found to be shorter on plant parts with a thin epidermis for the congeneric *Orius strigicollis* (Poppius) (Yu *et al.*, 2021). Thus, females may prefer sites that allow them to minimize the time spent ovipositing, reducing the risk of predation during this act. Another possible reason for the difference found between species may be the increased fitness benefits gained by *O. laevigatus* when feeding on floral resources that would eventually occur in the youngest plant parts, compared to *O. majusculus* (Oveja *et al.*, 2012). Both *Orius* predators in this study avoided ovipositing in the young flowers and leaves, possibly due to the high trichome density in these tissues (Stavrinides & Skirvin, 2003). Similarly, *Orius* predators avoid ovipositing in the young and trichome‐dense parts of soybean (Isenhour & Yeargan, 1982; Armer *et al.*, 1998) and cucumber (Jacobson, 1995) plants. However, it should be noted that pollen availability in the young flowers during our study was limited, which may explain the lack of oviposition from *Orius* predators in proximity to flowers.

No differences were found in the foraging behavior and predation on different plant strata between *O. laevigatus* and *O majusculus*. These results contrast the clear differences found between the species in within‐plant oviposition preferences. Food sources were never depleted at the end of the experiment in none of the offered strata, thus foraging for new food sources does not explain the lack of preference shown. The high mobility of *Orius* adults in conjunction with the relatively short length of chrysanthemum plants (∼60 cm) may have allowed them to move along the plant stem several times during the day, thus allowing them to exploit all available food sources freely, masking any underlying strata preferences. Indeed, the different spatial preferences between the two predators have been observed before on much taller plants in greenhouses that may grow several meters high, such as sweet pepper (van Schelt *et al.*, 2002) and cucumber (Ramakers & O'Neill, 1999). Furthermore, equal within‐plant foraging preferences were found also on flowering chrysanthemum plants in our study, contrary to our initial expectations. However, flowers in this experiment were probably not mature enough and pollen production was limited, which may explain this lack of preference for the upper plant strata by *Orius*.

Oviposition preferences of *O. laevigatus* on flowering chrysanthemum plants were found to be plastic and depended on the spatial availability of animal prey. Eggs were more frequently found in the lower plant strata when sentinel prey was also offered on older leaves, compared to when animal prey was available off‐plant. In contrast, when offering sentinel prey on younger plant tissue, no effect was found on the oviposition sites preferred by females. This is likely due to the high density of trichomes in the upper third of the chrysanthemum plants (Stavrinides & Skirvin, 2003), which may physically inhibit oviposition by *Orius* as discussed above (Lundgren *et al.*, 2008). While *Orius* predators are known to preferentially oviposit on plants that harbor sufficient arthropod prey (Venzon *et al.*, 2002; Seagraves & Lundgren, 2010), suitable plant characteristics may override the importance of animal prey availability for these predators when choosing oviposition sites (Groenteman *et al.*, 2006; Seagraves & Lundgren, 2010; Lundgren, 2011). However, in a previous study we found that the presence of animal prey on leaves did not influence the oviposition preference of *O. laevigatus* on flowering gerbera plants, which consistently oviposited in mature flowers where ample floral foods were available (Mouratidis *et al.*, 2023a). Furthermore, *Orius* predators consume a plethora of animal prey, ranging in mobility from sessile (moth eggs, whitefly nymphs) to highly mobile (thrips, spider mites) that may change their within‐plant distribution in response to the presence of a predator (Coll & Izraylevich, 1997; Walzer *et al.*, 2009). Thus, we hypothesize that while the presence of sedentary animal prey may have a predictable effect on the foraging and oviposition preferences of *Orius*, predators may respond dynamically to mobile prey. Further research into the oviposition preferences of *Orius* predators in response to the spatial availability and quality of animal and floral resources is necessary to improve our understanding of the behavior of omnivorous predators.

Fecundity and egg‐hatch success of *Orius* females differed between species but not due to differently aged chrysanthemum stems offered to the predators as oviposition substrate. *Orius laevigatus* laid overall more eggs in the 14‐d study period than *O. majusculus*, consistent with previous studies (Mouratidis *et al.*, 2022). However, stem age and by extension epidermal thickness did not influence the oviposition rate of the females, contrary to our expectations. *Orius* generate force to insert their eggs in the plant tissue by providing leverage from their tarsi (Shapiro & Ferkovich, 2006; Yu *et al.*, 2021). Thus, we hypothesized that the increased energy needed by females to embed their eggs in the thicker tissue would result in a reduced oviposition rate. Perhaps the difference in epidermal thickness was not profound enough to allow for such differences to manifest. For dragonflies that oviposit endophytically, tissue stiffness was shown to have a negative effect on oviposition rate (Lambret *et al.*, 2015). Significantly more eggs of *O. majusculus* successfully hatched compared to *O. laevigatus* across both stem ages tested. This result contrasts previous findings showing similar and high hatch rates for both predators on bean pods (Mouratidis *et al.*, 2022); however, the high suitability of bean pods as an oviposition substrate for *Orius* predators may have masked underlying differences between the species. Egg‐hatch success was not significantly affected by stem age; however, lower hatch rate on older stems was observed for both predators (Fig. 4B). During the experiments, *Orius* eggs were occasionally found only partly inserted in the plant tissue, while in other cases older plant stems wounded from oviposition responded by forming callus, partly ejecting the eggs (personal observations). Similarly, disorganization of the plant tissue due to oviposition by *O. laevigatus* was also observed and described on tomato plants (De Puysseleyr *et al.*, 2011). We hypothesize that older stem tissue may be physically tougher to penetrate and react stronger to wounds from oviposition, resulting in a reduced egg‐hatch rate.

Neonate nymphs of both *Orius* species could not complete their juvenile development feeding only on chrysanthemum plant tissue, and exhibited a maximum longevity of 8 d. *Orius laevigatus* nymphs survived significantly longer when they had access to young chrysanthemum tissue. Young chrysanthemum leaves are known to be more nutritious for arthropods than mature ones (Kielkiewicz & van de Vrie, 1990), due to their high nitrogen and soluble protein levels, which may explain the longer survival of *O. laevigatus* nymphs in our experiment. Similarly, *Orius albidipennis* nymphs survived longer on cotton leaves with higher nitrogen levels (Groenteman *et al.*, 2006). Anthocorids select oviposition sites using their rostrum rather than their ovipositor (Evans, 1976; Lundgren *et al.*, 2008), which suggests that they assess the nutritional value of the plant tissue for the initial development of their offspring, and may even actively defend their preferred sites from intraspecifics (Groenteman *et al.*, 2006). The results of our study corroborate this hypothesis, as *O. laevigatus* laid most of its eggs in the tender and nutritious plant parts of chrysanthemum, which ensured longer nymphal survival and slightly increased egg‐hatch rate. In contrast, the survival of *O. majusculus* nymphs was not affected by the age of plant tissue offered for plant feeding, suggesting that this species gains limited nutrition from chrysanthemum plant vascular sap.

There is now a growing body of literature on the oviposition behavior of *Orius* predatory bugs. These predators appear to select plants based on anatomical and morphological characteristics upon landing on them, and volatiles are thought not to be involved in this process (Lundgren *et al.*, 2009). Then, within‐plant sites are selected based on their epidermal thickness and trichome density (Groenteman *et al.*, 2006; Lundgren & Fergen, 2006; Lundgren *et al.*, 2008; Pascua *et al.*, 2019; Zhang *et al.*, 2021). These preferences appear to positively correlate with increased offspring performance, based on our findings and previous research (Groenteman *et al.*, 2006; Lundgren *et al.*, 2008). Within‐plant oviposition preferences have previously been anecdotally related to the proximity of animal and flower resources (Tawfik & Ata, 1973; Isenhour & Yeargan, 1982; van den Meiracker & Sabelis, 1993; Pascua *et al.*, 2019); however, the relative importance of these two resources for ovipositing females has not been elucidated. Here, we show that the within‐plant spatial availability of animal food influences the oviposition preference of *O. laevigatus* on chrysanthemum plants. This finding may lead to direct applications, whereby providing supplemental food sources low in the plants’ canopy, a common practice during the preventive establishment of these predators (Pijnakker *et al.*, 2020), may reduce the number of eggs deposited on young tissue that may be removed due to pruning or harvesting (van den Meiracker & Sabelis, 1993; Bekendam *et al.*, 2023). Furthermore, to the best of our knowledge, this is the first study including more than a single species of *Orius* while studying their oviposition behavior, showing that closely related species may differ in their use of host plants. We believe multispecies studies are important, as they may ultimately lead to new hypotheses on the oviposition behavior of omnivorous predators, while also providing insights relevant for their improved application in the field.

## Disclosure

The authors declare that no conflict of interests exists.

## Supporting information




**Fig. S1** The experimental setup of the within‐plant oviposition preference of *Orius* predators on (A) vegetative or (B) flowering chrysanthemum plants. A strip of *Artemia* cysts is attached along the main stem, providing a food source to the *Orius* predators (C) across the stem.
