# Supplementary material for: Differences in within‐plant oviposition preferences and immature survival between Orius predators and the importance of spatial availability of prey
Source: Insect Sci. 2024 Oct 31;32(4):1415–26. doi: 10.1111/1744-7917.13465 (PMC12369455; doi:10.1111/1744-7917.13465)
Supplement: Supplementary file 1 — Fig. S1 The experimental setup of the within‐plant oviposition preference of Orius predators on (A) vegetative or (B) flowering chrysanthemum plants. A strip of Artemia cysts is attached along the main stem, providing a food source to the Orius predators (C) across the stem. [file INS-32-1415-s001.docx]

**Differences in oviposition preferences between *Orius* predators and the importance of prey spatial availability**

## **Supplementary information**


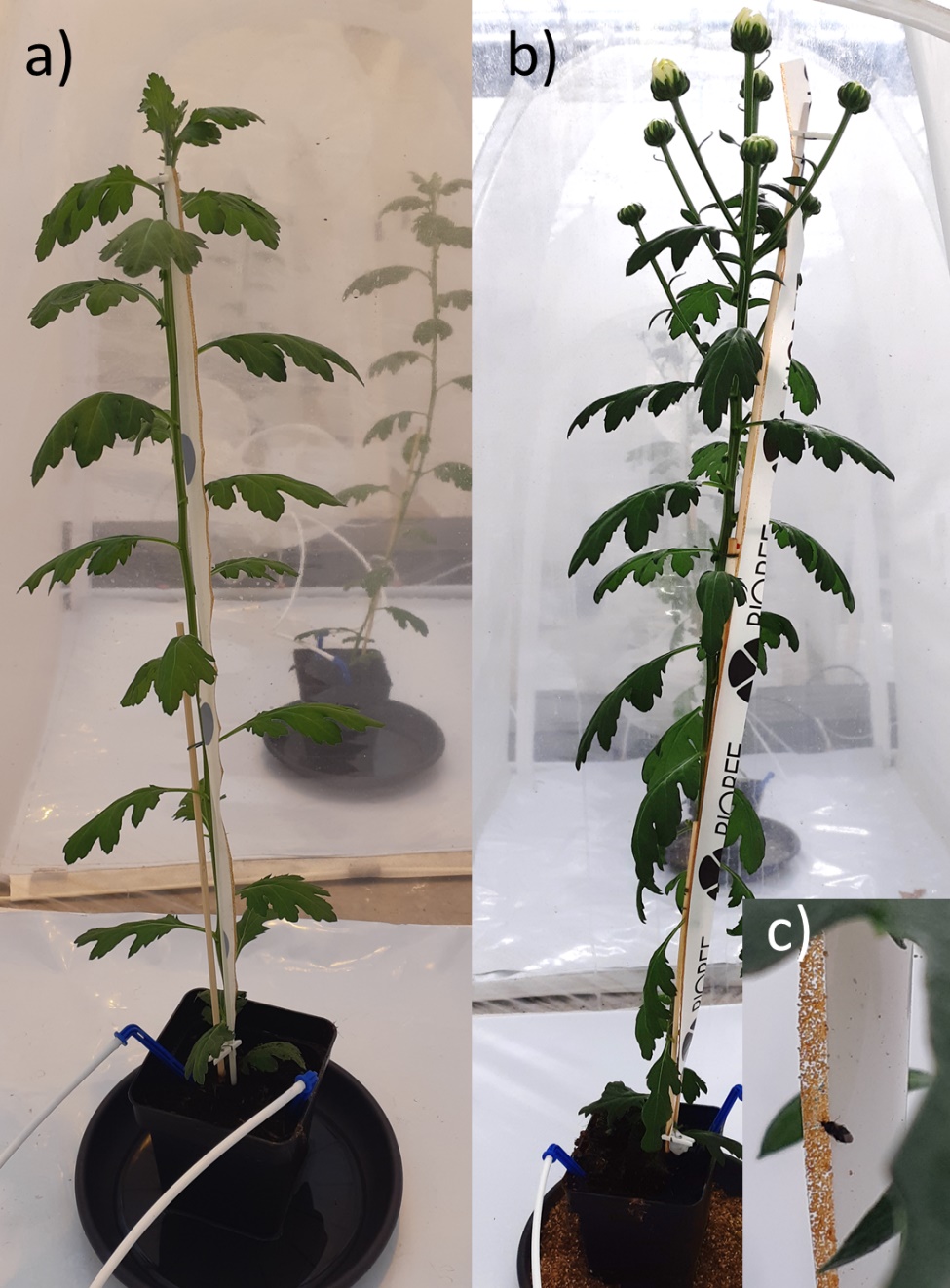


Fig. S1 The experimental setup of the within-plant oviposition preference of *Orius* predators on a) vegetative, or b) flowering chrysanthemum plants. A strip of *Artemia* cysts is attached along the main stem, providing a food source to the *Orius* predators (c) across the stem.
